# Giant lobelias exemplify convergent evolution

**DOI:** 10.1186/1741-7007-8-3

**Published:** 2010-01-14

**Authors:** Thomas J Givnish

**Affiliations:** 1 Department of Botany, University of Wisconsin, Madison, WI 53706, USA

## Abstract

Giant lobeliads on tropical mountains in East Africa and Hawaii have highly unusual, giant-rosette growth forms that appear to be convergent on each other and on those of several independently evolved groups of Asteraceae and other families. A recent phylogenetic analysis by Antonelli, based on sequencing the widest selection of lobeliads to date, raises doubts about this paradigmatic example of convergent evolution. Here I address the kinds of evidence needed to test for convergent evolution and argue that the analysis by Antonelli fails on four points. Antonelli's analysis makes several important contributions to our understanding of lobeliad evolution and geographic spread, but his claim regarding convergence appears to be invalid. Giant lobeliads in Hawaii and Africa represent paradigmatic examples of convergent evolution.

## Commentary

Many of us were inspired, at an early age, by Hedberg [1] and Carlquist [2] describing the bizarre, unbranched shrubs with massive leaf rosettes that dominate equatorial alpine zones in many parts of the world. Such plants share a highly unusual climate - 'summer every day, winter every night' [1] - and a highly unusual growth form characterized by large woody stems, extensive water-storage tissue in the stem pith and large leaves that persist on the stem after they die and, while alive, often curl around the terminal bud at night or impound rainwater surrounding that bud. These traits appear to be adaptations to nightly frosts, insulating vulnerable buds and water-storage tissue from a few hours of low temperatures, facilitating relatively rapid growth away from the ground where the highest diurnal fluctuations in temperature occur and providing moisture to permit photosynthesis during morning droughts when the sun is up but the ground still frozen [3-5]. These rosette shrubs - belonging to the families Asteraceae, Lobeliaceae, Valerianaceae and Bromeliaceae - appear to be striking manifestations of convergent evolution, of the independent acquisition of similar adaptations to similar environments by members of distantly related lineages.

This story became even more remarkable when it was later confirmed, by phylogenetic analyses of DNA sequences [6-8], that rosette shrubs had evolved independently at least three times in Asteraceae alone, including *Dendosenecio *of tribe Senecioneae on the volcanoes of tropical East Africa, *Espeletia *of tribe Heliantheae subtribe Melampodiinae in the northern Andes, and the silverswords (*Arygyroxiphium*) of tribe Heliantheae subtribe Madiinae of the Hawaiian Islands. DNA sequences - especially of non-coding regions with little or no possible selective value - have an advantage for analysing phylogenetic relationships among species invading extreme environments and/or undergoing adaptive radiation on islands or island-like habitats. In such situations, morphology is likely to often be a poor guide to ancestral relationships, given the strong selection on members of different (but perhaps closely related) lineages to converge in form and physiology in an extreme habitat, and on members of a given lineage to diverge from each other after invading a new area [9].

Like Asteraceae, Lobeliaceae (about 1200 species) is composed primarily of herbaceous plants from temperate and subtropical areas. However, in *Lobelia *and several closely related genera, woodiness has evolved in > 450 species, and 'giant' pachycaulous rosette shrubs grow in alpine areas and subalpine bogs of East Africa, South Asia, Polynesia, Hawaii and the Brazilian Shield and in semi-arid areas of Chile; unbranched or sparsely branched species with somewhat more slender stems occur in montane forests in these areas and the Andes. Hedberg and Carlquist suggested that giant woody lobeliads from high elevations in East Africa and Hawaii were convergent on Afroalpine *Dendrosenecio*, Andean *Espeletia*, and Hawaiian *Argyroxiphium*. Carlquist [10,11] argued that these giant rosettes and related woody species in both areas evolved ultimately from herbaceous ancestors, while Mabberley [12,13] instead argued that the pachycaul habit was primitive, with giant rosette shrubs and allied woody species with more slender stems forming a single worldwide lineage, with high-elevation species being derived from less specialized forms at mid elevations and with herbaceous *Lobelia *being derived independently from different woody sublineages. To complicate matters further, several botanists had proposed that the endemic Hawaiian lobeliads were not all closely related to each other but, instead, represented the product of up to five long-distance dispersal events (see review by Givnish *et al*. [14]).

Over the past 16 years, several groups have attempted to use molecular data to analyse relationships and patterns of adaptive evolution and geographic diversification among giant woody lobeliads and allied species. Knox *et al. *[15] used plastid DNA restriction-site data to show that woody tetraploid species of *Lobelia *and related genera from East Africa, Hawaii, Polynesia, the Bonin Islands, South Asia and Chile formed a clade and that they were derived from herbaceous ancestors. Knox and Palmer [16] used plastid DNA sequences to show that the giant and woody species of *Lobelia *from East Africa formed a clade, with the exception of a few Brazilian species derived via trans-Atlantic dispersal. Their phylogeny implied that the most specialized, giant-rosette shrubs of the Afroalpine zone (for example, *Lobeliaceae deckenii*, *L. rhynchopetalum, L. telekii, L. wollastonii*) evolved from ancestral, unbranched mid-elevation species with more slender stems, similar to present-day *L. giberroa *- supporting one of Mabberley's proposals. Givnish *et al. *[14] used plastid sequence data to show that the Hawaiian woody lobeliads are monophyletic; that they colonized the Hawaiian archipelago 13.0 to 13.6 million years ago, based on independent calibrations based on the ages of islands to which present-day species are restricted or the ages of fossil Asterales; that the giant species of *Lobelia *sect. *Galeatella *of subalpine bogs and grasslands are apparently derived from mid-elevation ancestors with more slender stems, similar to those of present-day *Clermontia, Cyanea *and *Delissea*; and that their closest relatives are the woody lobeliads of East Africa, Polynesia/Bonin Islands and South Asia.

Antonelli [17] has now addressed several of these same questions - and, most notably, the question of whether giant woody lobeliads are the product of convergent evolution or are instead all closely related - in a new phylogenetic study based on the most comprehensive set of lobeliads sequenced to date (101 spp.). This investigation has much to recommend it. First, it documents an 'Out of Africa' scenario for the origin of Lobeliaceae, based on the large number of small annual herbs native to Africa that form a grade just above the basal node of the family. Second, it confirms the hypothesis advanced by Knox *et al*. [15] that woody lobeliads evolved from herbaceous ancestors. Third, it confirms and extends findings by Knox, Givnish and their colleagues that lobeliads have repeatedly dispersed across the Atlantic and Pacific Oceans, with the new data supporting additional movements to the Neotropics, various parts of Oceania and southeast Asia. The production of thousands of dust-like, wind-dispersed, seeds by most lobeliads doubtless accounts for most of these long-distance dispersal events. Calibration of Antonelli's molecular phylogeny against fossils and various geologic milestones to form a timeline of lobeliad evolution (see also [14]) excludes continental drift as a possible explanation for these disjunctions. Fourth, based on an excellent sampling of genera and geographic regions occupied, Antonelli provides the first nearly complete view of geographic diversification across the family. A major finding is the SCBL clade (*Siphocampylus, Centropogon, Burmeistera, Lysipomia*), which comprises half the family and is endemic to the Neotropics. This clade is especially diverse in the northern Andes, which has undergone massive uplift over the past 10-20 million years and may, in so doing, have driven high rates of speciation in several other plant groups (for example, *Espeletia, Fuchsia*, Bromeliaceae: Tillandsioideae, Rubiaceae) [18-22]. The SCBL clade is embedded in a broader New World clade, including taxa from North America, the Caribbean, Central America and temperate Chile, as well as a few taxa representing long-distance dispersal events to the Pacific and Asia. This New World clade is sister to the clade consisting of the Old World woody lobeliads; strong support for the latter confirms the clade sister to and including *L. nicotianifolia *found by Givnish *et al. *[14]. Further investigation of the New World clade should pay rich dividends, given its extraordinary diversity of growth forms and geographic distributions, and what appear to be independent origins of the woody habit in the Caribbean (for example, *L. portorescensis*, *L. vivaldii*), temperate Chile (for example, *L. polyphylla*), and the montane Neotropics (for example, *Burmeistera, Centropogon*).

Finally, and surprisingly, Antonelli uses his phylogeny to argue that the giant woody lobeliads of Hawaii and East Africa are *not *examples of convergent evolution. This is, to me, a baffling claim - and yet one that Antonelli clearly feels is vitally important, given that 'testing' this claim forms the frame for his entire paper. This claim, I will now show, cannot be supported by the data and rationale provided.

There are four fundamental flaws in Antonelli's claim. First, his definition of convergence for giant lobeliads is overly restrictive and sets up a straw man:

'These results confidently show that lobelioid species commonly called "giant" are very closely related and have not developed their giant form from herbaceous ancestors independently...According to some early authors [Hedberg, Fries & Fries, Mabberley], convergence from herbaceous plants into tall treelets would have occurred independently in different mountain systems in response to similar tropical alpine climates consisting of nightly frosts and rapid temperature fluctuations.'

Why restrict convergence on the giant habit to those cases in which herbs were the immediate ancestors? Would convergence on that highly distinctive growth form be any less convergence if the ancestral form were not herbaceous, but some other life form instead? Obviously, no. One of the 'early' authors whom Antonelli cites is Mabberley [12]. Not only does Mabberley not speak of an herbaceous ancestor, he outlines a hypothesis in which the ancestral taxa from which giant forms adapted to high elevations in the tropics are derived from less specialized *woody *species; in which herbaceous species occasionally evolve from woody species; and, most importantly, in which all African, Hawaiian and South Asian woody lobeliads are considered part of *Lobelia *section *Rhychopetalum*. More broadly, almost all authors positing specific ancestors for the Hawaiian lobeliads named only woody species (see [14]). In sum, Antonelli's artful restriction of convergence on the giant growth form to only those cases with different herbaceous ancestors is overly restrictive and logically unnecessary, and does not correspond to the historical use of the idea by most authors, including those actively working on the African and Hawaiian lobeliads today (see [14-16]). Antonelli, in using this inappropriate definition, also chose to ignore the conclusions of Mabberley, who had - in the absence of molecular data - pointed to the independent origins of giant lobeliads in Africa and Hawaii from closely related woody progenitors.

Second, Antonelli's phylogeny lacks sufficient resolution to evaluate whether giant species from Hawaii and Africa (or elsewhere) form a clade or not. While he found that woody species from Africa, South Asia, Polynesia, and the Bonin and Hawaiian Islands form a clade - consistent with findings by Givnish *et al*. [14] and Knox *et al. *[15] - Antonelli's phylogeny has a seven-way polytomy that prevents assessment of relationships among the different geographic groups and, for the African and Hawaiian taxa, of relationships within such groups. Claiming that the African and Hawaiian giant lobeliads are not convergent simply because they '*all derive from a single ancestor' *is meaningless. All monocots share a single ancestor. However, does this mean that more than 20 apparent origins of fleshy fruits and of net venation [23] are not, in fact, independent? No. Among such origins are intercalated many groups with the capsular fruits and parallel venation ancestral to monocots, allowing the inference that monophyletic groups characterized by fleshy fruits or net venation represent independent origins. So, too, with woody lobeliads - only a minority of species have the giant growth form adapted to tropical alpine conditions, and detailed analyses of phylogenetic relationships within the Hawaiian taxa show that they are restricted to *Lobelia *sect. *Galeatella *and are embedded deep inside the Hawaiian clade, with non-giant woody species forming the remainder of that clade [14]. Similarly, in East Africa the alpine lobelias with the most extreme giant-rosette growth-forms are embedded in a broader group containing less extreme forms [16]. There is no doubt, given the description of the giant growth form by Antonelli, the specific species illustrated, and the thermal adaptive values he ascribes to the growth form, that he meant to restrict the usage of 'giant' to alpine and subalpine giant rosettes and not apply it more broadly to species with more slender stems, found in forests at lower elevations.

Third, it is misleading for Antonelli - in reconstructing ancestral character-states - to have overlaid all growth forms on the phylogeny *except *the very giant-rosette growth form whose independent origin(s) nominally are being assessed. Reconstruction of a nanophanerophyte (tree or treelet < 3 m tall) as the ancestor of the N4 clade says nothing about the distribution of origin(s) of the giant-rosette form within that clade. Overlaying the giant-rosette growth-form and reconstructing its ancestral occurrence is vital for *any *evaluation of evolutionary convergence.

Finally, in assessing the possibility of evolutionary convergence or stasis - of whether a particular trait has evolved independently in different lineages under similar ecological conditions, been maintained under such conditions and/or lost upon invasion of different conditions - it is equally vital to overlay such conditions on a phylogeny and reconstruct their ancestral states. This was not done by Antonelli, but should be considered best practice. Indeed, where possible, a formal analysis of correlated evolution of growth forms and environments should be conducted (for example, see [23]). Givnish *et al. *[14] showed that the ancestral environments of the giant species of *Lobelia *sect. *Galeatella *were alpine and subalpine bogs in Hawaii.

Thus, while Antonelli [17] has made fundamental contributions to our understanding of lobeliad evolution, I must take exception to his claim that African and Hawaiian giant lobeliads are not examples of convergent evolution. Manifestly, they are strikingly similar to the independently evolved species of *Dendrosenecio *in Africa, *Espeletia *in South America, and *Argyroxiphium *in Hawaii and both groups of giant lobeliads also represent independent origins from woody ancestors. All of these groups share a distinctive tropical-alpine climate, as well as several distinctive morphological traits. Many of the latter have been demonstrated to be of adaptive value in tropical-alpine climates. Therefore, the giant lobeliads represent examples - indeed, paradigmatic examples - of convergent evolution.

There remains the deeper question of whether origins judged independent, based on the preceding criteria, are truly independent. Might there be some developmental pathway or pattern of genetic variation in a broader lineage that makes the repeated origin of a particular trait more likely? Only detailed genetic studies can reveal whether the same genes and/or pathways are tapped in 'independent' origins. Such investigations have shown, for example, that melanic pelage has evolved in wholly different ways in rock pocket mice on black lava flows in Arizona versus New Mexico [24].

Future studies of lobeliad evolution could benefit from two new approaches that could foster research by the community as a whole. First, broad-scale phylogenetic studies should incorporate far more sequence data, to help resolve many of the very short branches in the lobeliad phylogeny (for example, those involving the divergences of the clades from Hawaii, Polynesia, the Bonin Islands and South Asia [14]). With next-generation DNA sequencing becoming cheaper and more powerful almost monthly, it should soon be possible to sequence whole-plastid genomes for scores of species. Eric Knox (personal communication) has such plans for several lobeliad genera and my colleagues and I hope to sequence plastomes for representatives of all Hawaiian genera in the immediate future. Second, it would be extremely helpful to sequence the entire genome of at least one lobeliad, to facilitate studies on the genetic basis for the extraordinary range of variation in several ecological significant traits seen across the family or in smaller groups (for example, fleshy fruits versus capsular fruits with wind-dispersed seeds; succulent versus non-succulent stems; insect- versus bird-pollinated flowers). A growing cadre of lobeliad ecologists and evolutionary biologists, and the broader evolutionary community, would greatly benefit from such studies.
